# Effectiveness and safety of XEN45 implant over 12 months of follow-up: data from the XEN-Glaucoma Treatment Registry

**DOI:** 10.1038/s41433-023-02642-5

**Published:** 2023-07-06

**Authors:** Francesco Oddone, Gloria Roberti, Sara Giammaria, Chiara Posarelli, Giorgio Ghirelli, Leonardo Mastropasqua, Luca Agnifili, Tommaso Micelli Ferrari, Vincenzo Pace, Paolo Nucci, Matteo Sacchi, Gianluca Monsellato, Romeo Altafini, Gianluca Scuderi, Andrea Perdicchi, Maurizio Uva, Carmela Carnevale, Giuseppe Covello, Maria Novella Maglionico, Antonio Fea, Michele Figus

**Affiliations:** 1 IRCCS - Fondazione Bietti, Rome, Italy; 2 Department of Surgical, Medical, Molecular Pathology and of Critical Care Medicine, University of Pisa, Pisa, Italy; 3 San Pietro Fatebenefratelli Hospital, Rome, Italy; 4 Ophthalmology Clinic, Department of Medicine and Aging Science, University G. D’Annunzio of Chieti-Pescara, Chieti, Italy; 5 Regional General Hospital F. Miulli of Acquaviva delle Fonti, Bari, Italy; 6 Department of Biomedical, Surgical, and Dental Sciences, University of Milan, Milan, Italy; 7 Eye Clinic, San Giuseppe Hospital - IRCCS Multimedica, Milan, Italy; 8 Ophthalmology Clinic, Dolo Hospital, Venezia, Italy; 9 Ophthalmology Unit, St. Andrea Hospital, NESMOS Department, University of Rome “Sapienza”, Rome, Italy; 10 University Hospital “Policlinico Vittorio Emanuele”, Catania, Italy; 11 Struttura Complessa Oculistica, Città Della Salute e Della Scienza di Torino, Dipartimento di Scienze Chirurgiche-Università Degli Studi di Torino, 10126 Torino, Italy

**Keywords:** Surgery, Optic nerve diseases

## Abstract

**Objectives:**

To evaluate the 1-year effectiveness and safety of the XEN45, either alone or in combination with phacoemulsification, in glaucoma patients.

**Methods:**

This multicentre, prospective, observational study included consecutive eyes of glaucoma patients from the Italian XEN-Glaucoma Treatment Registry (XEN-GTR) who underwent XEN45 alone or in combination with phacoemulsification, with at least 1 year of follow-up. Surgical success was defined as intraocular pressure (IOP) < 18 mmHg and ≥20% reduction from preoperative IOP, over 1 year of follow-up.

**Results:**

Two hundred thirty-nine eyes (239 patients) were analyzed, 144 (60.2%) eyes in the XEN-solo and 95 (39.8%) eyes in the XEN+Phaco groups. One hundred-sixty-eight (70.3%) eyes achieved overall success, without statistically significant differences between study groups (*p* = 0.07). Preoperative IOP dropped from a median (IQR) of 23.0 (20.0–26.0) mmHg to 14.0 (12.0–16.0) mmHg at month 12 (*p* < 0.001), with overall 39.9 ± 18.3% IOP reduction. The mean number of preoperative ocular hypotensive medications (OHM) was significantly reduced from 2.7 ± 0.9 to 0.5 ± 0.9 at month 12 (*p* < 0.001). Preoperative IOP < 15 mmHg (HR: 6.63; 95%CI: 2.61–16.84, *p* < 0.001) and temporal position of the surgeon (HR: 4.25; 95%CI: 2.62–6.88, *p* < 0.001) were significantly associated with surgery failure. One hundred-forty-six (61.1%) eyes had no intraoperative complications, whereas 91 (38.1%) and 56 (23.4%) eyes experienced at least one complication, respectively early (< month 1) and late (≥ month 1), all self-limiting or successfully treated without sequelae. Needling occurred in 55 (23.0%) eyes at least once during follow-up.

**Conclusion:**

Over 1-year follow-up, XEN45 alone or in combination with phacoemulsification, had comparable success rates and effectively and safely lowered IOP and the need for OHM.

## Introduction

Glaucoma includes a group of chronic and progressive optic neuropathies, characterized by retinal ganglion cells death, degeneration of their axons and consequent visual field (VF) damage [1].

The first therapeutical approach for glaucoma commonly is the use of topical hypotensive medications to control intraocular pressure (IOP). However, in some cases medical treatment may be insufficient [2, 3] and surgery becomes an option [4, 5].

The implantation of XEN45 (Allergan, an Abbvie company) is a filtering surgical procedure aimed at diverting the aqueous humor from the anterior chamber of the eye to the subconjunctival space. The ab-interno approach allows the XEN45 to be considered less invasive than traditional subconjunctival filtering procedures, sharing the same range of complications with the latter.

The effectiveness and safety of XEN45, either alone or in combination with phacoemulsification, have been evaluated before [6–12], mostly through retrospective studies or studies with a limited number of patients [13].

Although randomized clinical trials (RCTs) represent the best epidemiological design for evaluating efficacy and safety of new treatments and surgical procedures [14], the results of prospective registries can help health policy and the patient-tailored management [15], because they reflect the actual clinical care.

The XEN-Glaucoma Treatment Registry (XEN-GTR) prospectively collects real-world data from glaucoma patients who underwent XEN45 implant, in several Italian Centers [16], with the aim of analyzing different clinical aspects of this type of surgery [17].

The main purpose of this study was to evaluate the 1-year effectiveness and safety of XEN45, either alone or in combination with phacoemulsification, in patients with glaucoma in the XEN-GTR. Additionally, we assessed preoperative and intraoperative factors associated with surgery failure.

## Methods

### Design

This was a multicentre, prospective, and observational study conducted on 10 Italian Centers between January 2018 and December 2021. Local Ethics Committee approved the study protocol. In accordance with the Declaration of Helsinki, all patients gave written informed consent after having been fully informed about the details of the study purposes.

### Study participants

One eye of glaucoma patients with medically uncontrolled IOP, poor compliance or intolerance to therapy was consecutively enrolled. The inclusion criteria for the XEN-GTR were: (I) age >18 years; (II) clinical diagnosis of open angle glaucoma [18], [including primary open angle glaucoma (POAG), pseudoexfoliation, and pigmentary glaucoma] with indication of XEN45 alone or combined with phacoemulsification; (III) patients with history of failed filtering surgery; (IV) willingness to comply with the study protocol.

Patients diagnosed with secondary glaucoma (different from pseudoexfoliation and pigmentary glaucoma), active ocular inflammation, conjunctival alterations in the implant area, active iris neovascularization, intolerance or allergy to glutaraldehyde or porcine derivatives were excluded.

The stage of VF damage was classified as early (MD ≥ −6 dB), moderate (MD between −6 and −12 dB), and severe (MD ≤ −12 dB) [19].

Data from eyes included in the XEN-GTR within 1 year after surgery were retrieved and analyzed.

### Study implant and surgical technique

XEN45 is made of porcine gelatine crosslinked with glutaraldehyde and has an inner lumen of 45 µm, a 150 µm external diameter, and 6 mm length. At the beginning of the surgery, a dose of 0.1 ml of mitomycin C (MMC) at a concentration of 0.2 mg/ml was injected under the conjunctiva. With a 27-gauge preloaded injector, the XEN45 was implanted through the trabecular meshwork, to artificially connect the anterior chamber and the subconjunctival space. All XEN45 were implanted ab-interno by experienced glaucoma surgeons (one per Center), with at least 10 XEN45 implants in their surgical background. Given the real-world nature of the XEN-GTR, the surgical procedure was not standardized among study Centers. The choice regarding the position of the implant site and the type of surgery (standalone or combined) was left to the surgeon preferences, according to the characteristics of each eye.

### Study visits

The study protocol included a baseline and postoperative visits at day 1, month 1, month 3, month 6 and month 12. Each examination included: (I) IOP measured with Goldmann applanation tonometer (mean of 2 measurements or median of 3 measurements if the difference of the 2 exceeded 2 mmHg), (II) visual acuity in decimals, (III) record of the number of ocular hypotensive medications (OHM), (IV) record of complications and additional procedures. VF were tested at baseline and at months 6 and 12.

### Study groups

Two study groups were identified for the analysis: XEN-solo (eyes that underwent XEN45 alone) and XEN+Phaco (eyes that underwent XEN45 combined with phacoemulsification).

### Outcome measures

The primary outcome measure was the proportion of patients classified as success over 1 year of follow-up. Success was defined as postoperative IOP < 18 mmHg and IOP reduction from baseline ≥20%, with (qualified success) or without (complete success) OHM. Overall success was considered the sum of complete and qualified success. Failure was defined as an IOP ≥ 18 mmHg in two consecutive follow-up visits, <20% reduction of IOP from baseline at month 12, need for surgical revision and/or additional glaucoma surgery, or clinical hypotony (IOP < 6 mmHg and loss of visual acuity ≥2 lines, after month 1). IOP > 18 mmHg in the first follow-up month was not considered as failure.

In-clinic additional procedures (including needling, digital massage and sub-conjunctival mobilization) were not considered failure, similarly to other procedures undertaken during the follow-up.

### Statistical analysis

The statistical analysis was performed using open-source software R (version 3.6.0) [20], and the survival (version 3.2-7) [21] and survminer (version 0.4.9) [22] packages.

Descriptive statistics included mean and standard deviation (SD), or median and interquartile range (IQR) for continuous variables and number (%) for categorical variables. After using Shapiro–Wilk test to assess normal distributions, two-sided Student *t* test or Mann–Whitney U test was used to compare continuous variables, whereas categorical variables were analyzed with Chi-square or Fisher’s exact tests, as appropriate. Repeated measures ANOVA and Greenhouse-Geisser correction were used to assess the within group changes in IOP and number of OHM. To account for missing data, we additionally conducted a last observation carried forward (LOCF) analysis. Kaplan–Meier survival curves were used to assess the cumulative probability of success of surgery. In the survival analysis, patients were censored if their follow-up ended before the failure criteria were met. A conditional Cox hazard model was used to test the association between preoperative and intraoperative factors with surgery failure on both univariable and multivariable basis. Among the preoperative factors, sex, age, right/left eye, glaucoma type, preoperative IOP, VF damage and number of baseline OHM were included. The intraoperative factors analyzed were surgery type, position of the surgeon, implant site, type of viscoelastic and intraoperative complications. The validity of the proportional hazards’ prerequisites was assessed with the Martingale residuals method. A *P* value of <0.05 was considered statistically significant.

## Results

Among the 257 eligible eyes, 15 were excluded because of missing of any follow-up data and 3 because of missing preoperative IOP data. A total of 239 eyes (239 patients) from 9 Centers were included. The XEN alone group included 144 (60.2%) eyes and the XEN+Phaco one included 95 (39.7%) eyes. Seventy-three patients were lost to follow-up before month 12.

### Preoperative and procedure characteristics

Table 1 summarizes the main preoperative demographic and clinical characteristics of the study eyes. In the overall study sample, median (IQR) age was 72.0 (65.0−78.0) years, 106 (44.3%) were women and 219 (91.6%) were diagnosed with POAG. The median (IQR) VF mean deviation (MD) was −11.0 (−18.0 to −5.2) dB and according to VF damage, 56 (23.4%), 63 (26.4%), and 103 (43.1%) eyes were classified as having early, moderate, and severe glaucoma, respectively.

**Table 1:** Baseline main demographic and clinical characteristics.

	Total (*n* = 239)	XEN (*n* = 144)	XEN+Phaco (*n* = 95)	*P*
Age, median (IQR)	72.0 (65.0–78.0)	73.0 (62.0–79.0)	72.0 (68.0–76.5)	0.54
Gender Female	106 (44.3%)	67 (46.5%)	39 (41.0%)	0.48
Ethnicity				
Caucasian	239 (100.0%)	144 (100.0%)	95 (100.0%)	–
BCVA, median (IQR)	0.5 (0.2–0.8)	0.7 (0.4–0.9)	0.4 (0.1–0.6)	<0.001
IOP, median (IQR)	23.0 (20.0–26.0)	24.0 (20.7–26.0)	23.0 (20.0–26.0)	0.28
N of OHMs, median (IQR)	3.0 (2.0–3.0)	3.0 (2.0–3.0)	3.0 (2.0–3.0)	0.06
Type of Glaucoma				
				0.29
POAG	219 (91.6%)	133 (92.3%)	86 (90.5%)	
PXFG	16 (6.7%)	8 (5.5%)	8 (8.4%)	
Steroid induced	3 (1.2%)	3 (2.0%)	–	
Not reported	1 (0.4%)	–	1 (1.0%)	
Lens Status				
				<0.001
Phakic	130 (54.4%)	35 (24.3%)	95 (100.0%)	
Pseudo-phakic	109 (45.6%)	109 (75.7%)	–	
Previous Procedures				
				<0.001
None	124 (51.8%)	33 (22.9%)	94 (98.9%)	
Phaco	109 (45.6%)	109 (75.7%)	–	
SLT/ALT^a^	5 (2.1%)	4 (2.7%)	1 (1.0%)	
Other	4 (1.6%)	4 (2.7%)	–	
MD (dB), median (IQR)	−11.0 (−18.0 to −5.2)	−12.0 (−18.0 to −6.0)	−10.0 (−16.0 to −5.0)	0.22
VF Severity				
				0.19
Better than −6 dB	56 (23.4%)	32 (22.2%)	24 (25.3%)	
−6 to −12 dB	63 (26.4%)	32 (22.2%)	31 (32.6%)	
Worse than −12 dB	103 (43.1%)	67 (46.6%)	36 (37.9%)	
Not reported	17 (7.1%)	13 (9.0%)	4 (4.2%)	
C/D Ratio, median (IQR)	0.7 (0.6–0.8)	0.7 (0.6–0.8)	0.7 (0.6–0.8)	0.46
Position of the Surgeon				
				0.06
Superior	161 (67.3%)	104 (72.2%)	57 (60.0%)	
Temporal	78 (32.6%)	40 (27.7%)	38 (40.0%)	
Position of the XEN45 implant				
				<0.001
Superior-nasal	217 (90.8%)	129 (89.6%)	88 (92.6%)	
Superior-temporal	4 (1.6%)	4 (2.7%)	0 (0.0%)	
Superior	4 (1.7%)	1 (0.7%)	3 (3.2%)	
Nasal	11 (4.6%)	9 (6.2%)	2 (2.0%)	
Temporal	1 (0.4%)	0 (0.0%)	1 (1.0%)	
Not reported	2 (0.8%)	1 (0.7%)	1 (1.0%)	
Viscoelastic type				
				<0.001
Cohesive	213 (89.1%)	122 (84.7%)	91 (95.8%)	
Dispersive	26 (10.9%)	22 (15.3%)	4 (4.2%)	

Categorical variables are reported as proportions and comparisons were made using a Fisher exact test. Continuous variables are reported in medians (interquartile range [IQR]) and compared with Wilcoxon test.

*BCVA* Best corrected visual acuity, *IOP* Intraocular pressure, *OHMs* ocular hypotensive medications, *POAG* primary open angle glaucoma, *PXFG* pseudoexfoliation glaucoma, *PACG* primary angle closure glaucoma, *SLT* selective laser trabeculoplasty, *ALT* Argon laser trabeculoplasty, *MD* Mean Deviation, *VF* Visual field, *C/D* Cup to Disc.

^a^Performed as standalone procedure or in patients with history of previous procedures.

The two study groups were similar in terms of demographic characteristics but the XEN+Phaco group showed worse visual acuity. XEN+Phaco was performed in 95 (73.1%) phakic eyes.

### Intraocular pressure

**Fig. 1:**
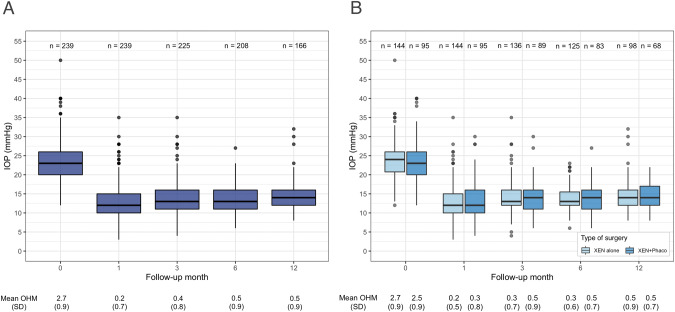
Distribution of intraocular pressure (IOP) and ocular hypotensive medications (OHM). At preoperative visit and through the 12 months of follow-up in the overall study sample (**A**) and in the XEN alone and the XEN+Phaco groups (**B**). Boxes represent the first and third quartiles and the horizontal lines across the boxes indicate the median.

**Fig. 2:**
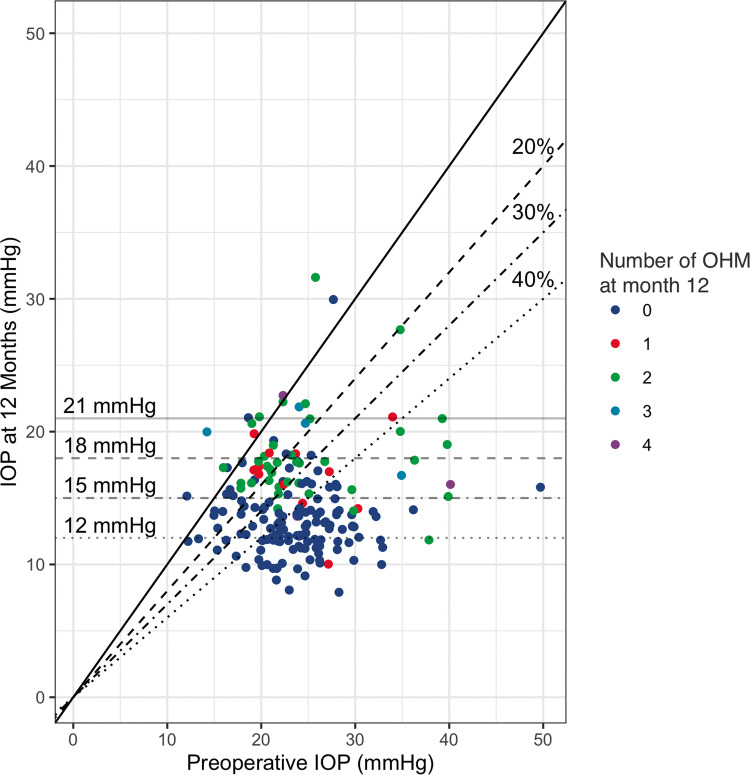
Scatter plot of the intraocular pressure (IOP) at preoperative visit vs. IOP at month 12, according to the preoperative IOP and number of ocular hypotensive medications. Each point represents one eye. The horizontal gray lines represent the IOP thresholds at month 12. The black lines represent the percentage levels of IOP reduction at month 12 compared with preoperative IOP. Eyes with missing IOP data are not shown. OHM ocular hypotensive medications.

The preoperative median (IQR) IOP was 23.0 (20.0−26.0) mmHg, which was significantly reduced to 12.0 (10.0−16.0) mmHg, 12.0 (10.0−15.0) mmHg, 13.0 (11.0−16.0) mmHg and 14.0 (12.0−16.0) mmHg at months 1, 3, 6, and 12, respectively (*p* < 0.001) (Fig. 1A). Using the LOCF, the analysis yielded the same values of IOP at month 12, with negligible differences in the total range of measurement (8−32 vs. 6−32 mmHg with LOCF). IOP did not significantly differ between study groups, at any time point (Fig. 1B). At month 12 the overall IOP reduction was 39.9 (18.3)%, with similar figures for the XEN alone [40.6 (17.8)%] and XEN+Phaco [39.9 (19.1)%] groups, *p* = 0.69. Figure 2 shows the IOP reduction as a function of preoperative IOP and the number of OHM at month 12. The number (%) of eyes that at month 12 achieved ≥ 20%, ≥30% and ≥40 % reduction from preoperative IOP were 139 (83.7%), 121 (50.6%) and 98 (41.0%). Using the LOCF approach the percentages were slightly lower: 175 (73.2%), 155 (64.8%) and 125 (52.3%) respectively. The final IOP was ≤21 mmHg, ≤18 mmHg, ≤15 mmHg and ≤12 mmHg in 159 (95.7%), 151 (90.9%), 112 (67.5%) and 58 (34.9%) eyes. With the LOCF, the respective figures were 228 (95.4%), 211 (88.2%), 160 (66.9%) and 84 (35.1%) eyes. Higher percentages of IOP reduction were achieved in eyes with higher preoperative IOP: from −6.7% (−16.7 to −9.1%) in eyes with IOP ≤ 15 mmHg to −61.1% (−65.2 to −56.2%) in eyes with IOP ≥ 35 mmHg (Supplementary Fig. 1).

### Ocular hypotensive medications

The mean number of OHMs significantly decreased from 2.7 (0.9) at baseline to 0.5 (0.9) at month 12 (*p* < 0.001) (Fig. 1A) with comparable results with the LOCF [0.6 (1.0) OHMs]. No significant differences were found between study groups at any time point (Fig. 1B).

At month 12, the number of OHM-free eyes (including topical and systemic medications), was 117 (71.5%), (67.7% with the LOCF). The number of eyes treated with 3 and 4 OHMs decreased from 62.0% at baseline to 2.6% at month 12 (Supplementary Fig. 2).

Moreover, the number (%) of patients additionally treated with oral systemic carbonic anhydrase inhibitors was significantly reduced from 84 (35.1%) to 2 (1.2%) at month 12 (*p* < 0.0001).

### Effectiveness

**Fig. 3:**
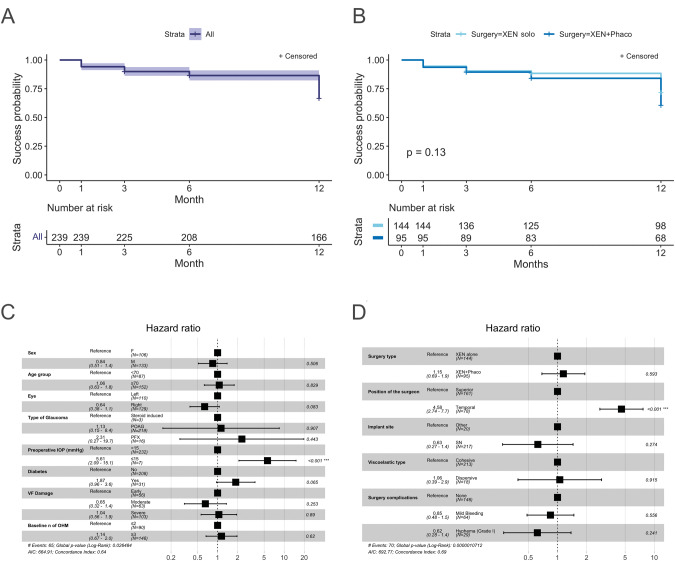
Kaplan–Meier survival curves. The success probability through the 12 months of follow-up, in the overall study sample (**A**) and in the XEN alone and the XEN+Phaco groups (**B**) and multivariate Cox proportional hazard ratios and 95% confidence intervals (CIs) for preoperative (**C**) and intraoperative (**D**) factors predictive of surgical failure. In survival curves, eyes lost to follow-up before month 12 and that did not meet the failure criteria were censored. F female, M male, PFX pseudoexfoliation glaucoma, IOP intraocular pressure, VF visual field, OHM ocular hypotensive medications, SN supero-nasal.

One hundred sixty-eight (70.3%) eyes achieved overall success over 1-year follow-up, with 139 (82.7%) of these eyes being classified as complete and 29 (17.2%) as qualified success. Overall success rates were comparable between the XEN alone [74.3%, with 92 (85.9%) being complete success] and the XEN+Phaco groups [64.2%, with 47 (77.0%) being complete success], (*p* = 0.07). Figure 3 shows the success probability for the overall study sample (Fig. 3A) and for the study groups (Fig. 3B). At 12 months, among 166 eyes, 119 were classified as overall success (71.7%). No statistically significant differences were found between study groups (Fig. 3B).

Among the 73 eyes lost to follow-up, 33 were classified as failures and the remaining 40 patients ended follow-up for other reasons [7 (17.5%) at month 3 and 33 (82.5%) at month 6]. Three out of the 33 (9.1%) eyes classified as failures before month 12, needed a XEN45 removal and reimplant whereas one (3.0%) showed clinical hypotony at month 3 which persisted at month 6 (last available follow-up).

In addition to the main analysis, we evaluated the survival and success probabilities with IOP thresholds of <15 mmHg and <21 mmHg (Supplementary Figure 3). For the <15 mmHg threshold, the success probability rates at month 12 were marginally higher in the XEN alone group (0.56; 95%CI: 0.48–0.66) compared with the XEN+Phaco group (0.43; 95% CI: 0.34–0.55), *p* = 0.048. No differences were found between the two study groups for the <21 mmHg threshold, which also showed higher success probabilities (0.74; 95%CI:0.67–0.81 and 0.76; 95%CI: 0.67–0.85 for the XEN alone and the XEN+Phaco groups respectively, *p* = 0.73).

A total of 71 (29.7%) eyes were classified as failure considering the whole sample: 1 eye developed clinical hypotony, 2 were reoperated (one eye underwent cyclophotocoagulation and one a second XEN45 implant), 5 needed surgical revision, while the remaining 63 (88.7%) eyes did not reach the required IOP reduction.

### Factors associated with surgery failure

A preoperative IOP < 15 mmHg was the only preoperative factor significantly associated with failure in both univariate (Hazard ratio, HR: 6.63; 95%CI: 2.61–16.84, *p* < 0.001) and multivariate analysis (HR: 5.61; 95%CI: 2.10–15.07, *p* < 0.001; Fig. 3C).

Among intraoperative factors, the temporal position of the surgeon was significantly associated with surgical failure in univariate analysis (HR: 4.25; 95%CI: 2.62–6.88, *p* < 0.001) and multivariate analysis (HR: 4.58; 95%CI: 2.74–7.64, *p* < 0.001; Fig. 3D).

### Safety

One hundred forty-six (61.1%) eyes had no intraoperative complications. Mild bleeding occurred in 64 (26.7%) eyes, hyphaema (Grade I) [23] in 29 (12.1%), and conjunctival tears in 1 (0.4%) eye.

Table 2 summarizes the postoperative complications and the additional procedures required during follow-up. Ninety-one (38.1%) eyes experienced at least one early complication and 56 (23.4%) eyes experienced at least one late complication. Forty-two (17.5%) eyes experienced more than one complication, either early or late after surgery. Choroidal detachment showed the highest frequency. Most complications were self-limiting or appropriately treated without impact on visual acuity or VF. Only 2 eyes experienced a serious complication (blebitis), which was adequately treated without sequelae. Other cases of hypotony maculopathy were not clinically significant.

**Table 2:** Patients who experienced postoperative complications and had additional procedures during the follow-up.

Post operative complications^a^			Additional procedures^b^	
	Early (<Month 1)	Late (≥Month 1)		
Corneal oedema	5 (2.1%)	9 (3.7%)	Needling	
Corneal dellen	0 (0.0%)	9 (3.7%)	Alone	27 (11.3%)
Shallow AC	17 (7.1%)	0 (0.0%)	+MMC	18 (7.5%)
Hyphaema	12 (5.0%)	0 (0.0%)	+ 5FU	14 (5.8%)
Iritis	0 (0.0%)	7 (2.9%)	+Corticosteroid	7 (2.9%)
AC flare	0 (0.0%)	3 (1.6%)	+Lidocaine	2 (0.8%)
XEN45 Blocked lumen	16 (6.7%)	12 (6.1%)	Manual subconjunctival mobilization	28 (11.7%)
Dislocated XEN45	0 (0.0%)	2 (0.8%)		
Conjunctival tear	0 (0.0%)	2 (0.8%)	Digital Massage	19 (7.9%)
Leak/dehiscence	5 (2.1%)	0 (0.0%)		
XEN45 erosion	0 (0.0%)	3 (1.2%)	XEN45 removal and reimplant	4 (1.6%)
Choroidal detachment	29 (12.3%)	2 (0.8%)		
Hypotony maculopathy	11 (4.6%)	5 (2.1%)	Cyclophotocoagulation	1 (0.4%)
Macular oedema	0 (0.0%)	7 (2.9%)		
Diplopia	0 (0.0%)	2 (0.8%)	Bleb drainage + Contact Lens	1 (0.4%)
Ptosis	0 (0.0%)	3 (1.2%)		
Serious complications			Other Procedures	
Blebitis	0 (0.0%)	2 (0.8%)	Laser for Luminal Obstruction	1 (0.4%)
Endophthalmitis	0 (0.0%)	0 (0.0%)	Conjunctival Suture	1 (0.4%)
Malignant glaucoma	0 (0.0%)	0 (0.0%)	IOL repositioning + reimplantation	1 (0.4%)
Angle closure	0 (0.0%)	0 (0.0%)	Second XEN45 implant	1 (0.4%)
Retinal detachment	0 (0.0%)	0 (0.0%)	AC refill with viscoelastic	1 (0.4%)

*MMC* Mitomycin-C, *5FU* 5-Fluorouracil, *IOL* Intraocular lens, *AC* Anterior chamber.

^a^Patients may have experienced single or multiple complications during follow-up.

^b^Eyes may have been treated with single, repeated or combined procedures during follow-up.

Ninety-two (38.5%) eyes required at least an additional procedure during follow-up. Needling was performed in 55 (23.0%) eyes at least once during follow-up for a total number of 89 needlings. Thirty eyes needed 1 needling, 18 eyes needed 2 needlings and 5 and 2 eyes needed 3 and 4 needlings, respectively. The number of eyes requiring needlings in the two study groups were comparable [32 (13.2%) and 23 (9.6%), *p* = 0.5].

Preoperative visual acuity significantly improved from 0.5 (0.3−0.8) to 0.8 (0.5−1.0) at month 12, *p* < 0.001. Improvement occurred in both the XEN solo [+0.1 decimals, *p* < 0.05] and the XEN+Phaco [+0.5 decimals, *p* < 0.001] groups.

In the subset of 156 eyes with available VFs at baseline and month 12, MD did not significantly change [−10 (−16 to −5) vs. −10 (−18 to −4) dB, *p* = 0.1] and the sub-analysis according to VF damage showed negligible differences (ranging from −1 to +1 dB).

## Discussion

This multicentre, prospective, and observational clinical study based on the XEN-GTR was carried out to assess the effectiveness and safety outcomes over 1 year after XEN45, alone or combined with phacoemulsification. Our results showed that both XEN45 and XEN+Phaco significantly lowered the IOP and reduced the number of OHM in patients with glaucoma.

At the end of the follow-up, more than half of the eyes achieved an IOP reduction >40% and a final IOP ≤ 15 mmHg. Furthermore, more than 70% of the eyes did not need any additional medical therapy. The success rate observed in our study was 70.7% for the whole sample. Although the criteria for defining success among different studies show heterogeneity, our findings are either consistent with those reported in previous studies [7, 10, 12, 24], or even suggestive of greater clinical success [6, 8, 25, 26].

The effectiveness of XEN45 alone and XEN+Phaco has been widely investigated (Supplementary Table) with conflicting results regarding the superiority of one surgery over the other [8, 27–30]. In agreement with previous studies [27–29], we did not find significant differences in terms of success rates and IOP reduction between the two surgeries. However, others reported higher success rates for the XEN alone [8, 30].

According to the definition of success adopted for our analysis, a preoperative IOP < 15 mmHg was associated with surgical failure in both univariate and multivariate analyses. Eyes classified as success showed preoperative IOP significantly higher than that observed in those classified as failure [24.0 (22.0–27.0) mmHg vs. 21.0 (18.0–25.0) mmHg, respectively, *p* < 0.001]. These results suggest that patients with low preoperative IOP should experience a small reduction of IOP. Indeed, eyes with preoperative IOP ≤ 15 mmHg achieved a median (IQR) IOP reduction of −6.7% (−16.7–9.1%) at months 12 (Supplementary Fig. 1) which is remarkably below the ≥20% IOP reduction cut-off. Nevertheless, at month 12, 85.7% of the eyes included in this subgroup were OHM free (Fig. 2), thus suggesting that XEN45 may be useful in reducing or avoiding OHM in these eyes. However, given the small number of eyes in this subgroup (*n* = 7), a larger sample is needed to draw stronger conclusions.

Since the definition of success is arbitrary and no consensus exists on an unambiguous definition [31], the rates of effectiveness from studies such as ours should be critically evaluated for the clinical implications that may arise from them. For example, a patient with an IOP of 28 mmHg at baseline and 19 mmHg postoperatively would be considered as surgical failure because the ≤18 mmHg IOP criteria for success is not met, despite the IOP reduction being >20%. For this reason, to explore the effect of different definitions of success, we evaluated different IOP threshold values, keeping the ≥20% IOP reduction. With the more stringent threshold of <15 mmHg, we found that the probability of success was still clinically relevant, being 56% and 43% for XEN alone and XEN+Phaco respectively. When <21 mmHg threshold was considered, the probability of success increased, as expected, to 74% and 76% (Supplementary Fig. 3).

In the current study, we observed a significant improvement in visual acuity at month 12 not only in the XEN+Phaco, as expected, but also in the XEN45 alone group. Although regression to the mean cannot be completely ruled out, improved visual function in glaucoma patients after surgical IOP reduction has been reported [32]. It has been hypothesized that in glaucoma, before retinal ganglion cell death takes place, reversibility of the underlying cellular dysfunction may occur [33]. This hypothesis has been supported by Ventura et al. [34], who found a disproportionate reduction in pattern electroretinogram amplitude compared to retinal nerve fibre layer thickness. Therefore, by reducing the IOP, partial functional restoration of impaired retinal ganglion cells might be possible, and this would potentially explain the improved visual function after surgery.

Concerning the safety profile, the incidence and type of complications are similar to current evidence [6–12, 24, 25, 27–30, 35–44] and the needling rate (23.0%) is consistent with previously reported rates that ranged between 2.4% and 43% [13].

The main strength of our study concerns its real-world and prospective design and the large number of eyes included, so that our results have a reasonable external generalizability to the Caucasian population. However, it is worth recalling that real-world studies have inherent limitations, including a non-randomized design, number of surgeons and variability in treatment decisions.

Regarding the latter, this study provided insight into the degree of VF damage in eyes eligible for XEN45 implantation. Good candidates for MIGS surgery are generally considered to be patients with mild-to-moderate glaucoma damage. In this study, glaucoma patients showed a median preoperative MD of −11 dB, with ~70% of patients having moderate-to-severe glaucoma. According to this finding, in real-world settings, surgeons are likely to choose alternative surgery options rather than traditional filtering surgery, even in patients with more damaged VF.

All the eyes included in the XEN-GTR were already medically treated before surgery, therefore, we have no data on untreated IOP. Moreover, the lack of standardized surgical procedures may be considered a relevant limitation of the study, and this may have influenced the results on effectiveness and overall success. Among the Centers, the surgical technique varied by implant site, position of the surgeon and the use of different types of medications during surgery. On the other hand, this heterogeneity allows us to explore the outcomes of different surgical approaches. For example, we unexpectedly documented that the relative risk of surgery failure was ~4 times higher when the surgeon was placed in the temporal position. In our sample the position of the surgeon was temporal in 27.7% of eyes in the XEN alone group and in 40.0% of eyes in the XEN+Phaco group (*p* = 0.028). We investigated whether the higher failure rates observed in surgeries performed from temporal position could be related to higher proportion of XEN+Phaco. Despite the percentages of the XEN+Phaco among the eyes operated superiorly and temporally were higher in the latter (being 35.4% and 48.7% respectively), the difference was not statistically significant (*p* = 0.06). In this regard, because of the small sample size in this sub-analysis that may have led to a type 2 error, we acknowledge that further investigation is needed to clarify this result.

The effect of standalone phacoemulsification on IOP has usually been reported to be mild in extent and transient [45–47] despite recently being described as having a higher IOP-lowering effect [48]. On the other hand, glaucoma filtering procedures combined with phacoemulsification may be associated with somewhat lower effectiveness than standalone filtering procedures, probably in relation to a higher grade of ocular inflammation and risk of scarring [49].

In this context, based on the data available in the XEN-GTR, we aimed at exploring whether XEN+Phaco had similar effectiveness compared to the XEN45 alone and we found, in agreement with previously published reports, that this is the case. Of course, an additional comparison group of glaucoma patients with uncontrolled IOP undergoing standalone phacoemulsification would be suited to explore the contribution of phacoemulsification to the overall effectiveness of the XEN+Phaco procedure. However, the XEN-GTR was designed to enrol only patients scheduled for XEN implantation with or without combined phacoemulsification and not patients undergoing standalone phacoemulsification. Nevertheless, in the future, analysis of long-term data from the XEN-GTR will allow to explore the influence of phacoemulsification performed after the XEN45 implantation on the bleb survival and on IOP control.

Another limitation of this study is the relatively short follow-up period. Glaucoma is a long-life chronic disease; therefore, 1-year follow-up may be insufficient to correctly assess the long-term outcomes of XEN45. However, the advantage of the prospective nature of the XEN-GTR is the opportunity to keep collecting follow-up data and to extend the analysis over a longer follow-up time in future works.

## Conclusions

The XEN45, either alone or in combination with phacoemulsification, significantly lowered the IOP and reduced the need of ocular hypotensive medications in glaucoma patients, although the effect of standalone phacoemulsification could not be ruled out with the combined procedure. Regarding safety, the incidence rate and type of complications are low in the first year after surgery and in line with those previously reported.

Our study did not find significant differences during follow-up between the eyes that underwent XEN alone and XEN+Phaco.

## Summary

### What was known before

- XEN45 implant have emerged as less invasive alternative to the traditional filtering surgery for glaucoma.
- Great part of information on efficacy and safety of XEN45 derived from retrospective case series or a limited number of prospective clinical trials.

### What this study adds

- The XEN-GTR is the first prospective multicentre real-world data collection with the aim of improving the understanding the role of this technique in glaucoma management.
- In the real life setting XEN45 is effective and safe. Implanted alone or in combination with phacoemulsification, XEN45 has comparable success rate.
- Lower preoperative IOP and surgeon position may influence the success rate of XEN45 implant.
- XEN45 may be a valuable surgical option also in glaucoma patients with moderate to advanced visual field damage.

### Supplementary information

Supplementary Table

Supplementary Figure Legends

Supplementary Figure 1

Supplementary Figure 2

Supplementary Figure 3

## Data Availability

The data collected and/or analyzed during the current study are available from the corresponding author upon reasonable request.
