# Supplementary material for: Effectiveness and safety of XEN45 implant over 12 months of follow-up: data from the XEN-Glaucoma Treatment Registry
Source: Eye (Lond). 2023 Jul 6;38(1):103–11. doi: 10.1038/s41433-023-02642-5 (PMC10764778; doi:10.1038/s41433-023-02642-5)
Supplement: Supplementary file 1 — Supplementary Table [file 41433_2023_2642_MOESM1_ESM.docx]

**Supplemental Table. Comparison of the clinical outcomes between the current study and the available evidence**

| **Study** | **Preop IOP**  **(mm Hg)** | **M12 IOP**  **(mmHg)** | **M12 IOP lowering** | **Mean preoperative OHM**  **(n)** | **Mean OHM, last visit**  **(n)** | **Needling Rates at last follow-up visit**  **n (%)** |
| --- | --- | --- | --- | --- | --- | --- |
| Galal et al (8) | 16.0 (4.0) | 12.0 (3.0) | 23^d^ | 1.9 (1.0) | 0.13 (0.11) | 4 (30.7) |
| Mansouri et al (10) | 20.0 (7.1) | 13.9 (84.3) | 31^d^ | 1.9 (1.3) | 0.5 (0.8) | 55 (36.9) |
| Reitsamer et al. (12) | 21.4 (3.6)^b^ | 14.9±4.5^b^ | − 6.5±5.3^b^ | 2.7±0.9^b^ | 1.1±1.2^b^ | 83 (41.1) |
| Fea et al. (14) | 23.9 (7.6)^b^ | 15.5±3.9^b^ | -7.4±7.9 | 3.0 (1.0) | 0.5±1.0^b^ | 79 (46.2) |
| Marcos-Parra et al. (20) | 19.1 (5.4)^b^ | NA | -6.7 (-12.9 to -0.5)^c^ | 2.5 (0.8) | 0.2±0.6^b^ | 13 (20.0) |
| Laborda-Guirao et al. (21) | 21.0 (5.2)^b^ | 14.7 (13.9 to 15.4)^c^ | -6.3 (-8.8 to -4.4)^c^ | 2.8 (2.7 to 3.0)^c^ | 1.1 (0.8 to 1.3)^c^ | 7 (8.8) |
| Gabbay et al (22) | 22.1±6.5^b^ | 15.4±5.9^b^ | -6.7±6.2^b^ | 2.77±1.1^b^ | 0.5±1.0^b^ | 57 (37.7) |
| Mansouri et al (23) | 20.0±7.5^b^ | NA | NA | 2.0±1.3^b^ | 0.6±0.9^b^ | 58 (45) |
| Grover et al. (24) | 25.1 (3.7)^b^ | 15.9±5.2^b^ | -9.1 (-10.7 to 7.5)^c^ | 3.5 (1.0) | 1.7^a^ | 21 (32.3) |
| Ibáñez-Muñoz et al. (25) | 22.3 (21.0-23.5)^c^ | 15.3 (14.3-16.3)^c^ | -7.3 (-9.7 to -5.0)^c^ | 3.0±1.0 | 1.2±1.2 | 19 (26.0) |
| Theilig et al. (26) | 24.5±6.7^b^ | 16.6 ± 4.8^b^ | NA | 3.0±1.1^b^ | 1.4 ± 1.5^b^ | 42 (42.0) |
| Hengerer et al. (27) | 32.2 (9.1)^b^ | 14.2±4.0 | 32.2^d,a^ | 3.1±1.0^b^ | 0.3±0.7^b^ | 67 (27.7)^f^ |
| Karimi et al (28) | 19.3 (6.0)^b^ | 14.3 (4.4)^b^ | -5.1±5.6^b^ | 2.6 (0.1) | 1.6 (0.5) | 106 (40.9)^g^ |
| Wanichwecharungruang & Ratprasatporn (29) | 21.6±4.0 | 15^a^ | 30.6^d,a^ | 2.1±1.4 | 0.5±0.7^b^ | 10 (17.5) |
| Subaşı et al (30) | 20.4±4.8^b^ | 15.0±1.9^b^ | -6.2±0.9^b^ | 3.1±1.0^b^ | 0.9±1.1^b^ | 13 (43.3) |
| Rauchegger et al (31) | 23.4±7.9^b^ | 14.6±3.6^b^ | 31(20-42)^d,e^ | 2.7±1.1^b^ | 1.0±1.2^b^ | 37 (62) |
| Reitsamer et al. (32) | 20.7 (5.1) | 14.8^a^ | -5.6^a^ | 2.5 (1.0) | 0.7 (1.0) | 78 (36.8) |
| Current study | 23.0 (20-26)^h^ | 14.0 (12-16)^h^ | 41.7(52.0 – 25.3) | 2.67 (0.9)^b^ | 0.57 (0.9)^b^ | 4 (17.4%) |

^a^Data about standard deviation was not provided.

^b^Mean (Standard deviation)

^c^Mean (95% confidence interval).

^d^Percentage.

^e^95% Confidence interval

^f^All the needling procedures were done between week 1 and months 3.

^g^Postoperative bleb needling or antimetabolite injection

^h^Median (Interquartile range).

Abbreviations:

IOP: Intraocular pressure; NOHM: Number of ocular hypotensive medications.
