# Supplementary material for: Effectiveness and safety of XEN45 implant over 12 months of follow-up: data from the XEN-Glaucoma Treatment Registry
Source: Eye (Lond). 2023 Jul 6;38(1):103–11. doi: 10.1038/s41433-023-02642-5 (PMC10764778; doi:10.1038/s41433-023-02642-5)
Supplement: Supplementary file 2 — Supplementary Figure Legends [file 41433_2023_2642_MOESM2_ESM.docx]

SUPPLEMENTARY FIGURES LEGENDS

**Supplementary Figure 1**. Distribution of the percentage of intraocular pressure (IOP) reduction according to preoperative IOP range, in the overall study sample (A) and in the XEN alone and the XEN+Phaco groups (B). Boxes represent the first and third quartiles and the horizontal lines across the boxes indicate the median.

**Supplementary Figure 2.** Distribution of the percentage of ocular hypotensive medications (OHM) at preoperative visit and month 12. Eyes with missing OHM data are not shown.

OHM = Ocular Hypotensive Medications

**Supplementary Figure 3.** Kaplan-Meier survival curves showing the success probability between XEN alone and the XEN+Phaco groups, according to different IOP threshold of success: <15 mmHg threshold (A), and <21 mmHg threshold (B). Eyes lost to follow-up before month 12 that did not meet the failure criteria were censored.
