# Supplementary figures and images for: Effectiveness and safety of XEN45 implant over 12 months of follow-up: data from the XEN-Glaucoma Treatment Registry
Source: Eye (Lond). 2023 Jul 6;38(1):103–11. doi: 10.1038/s41433-023-02642-5 (PMC10764778; doi:10.1038/s41433-023-02642-5)

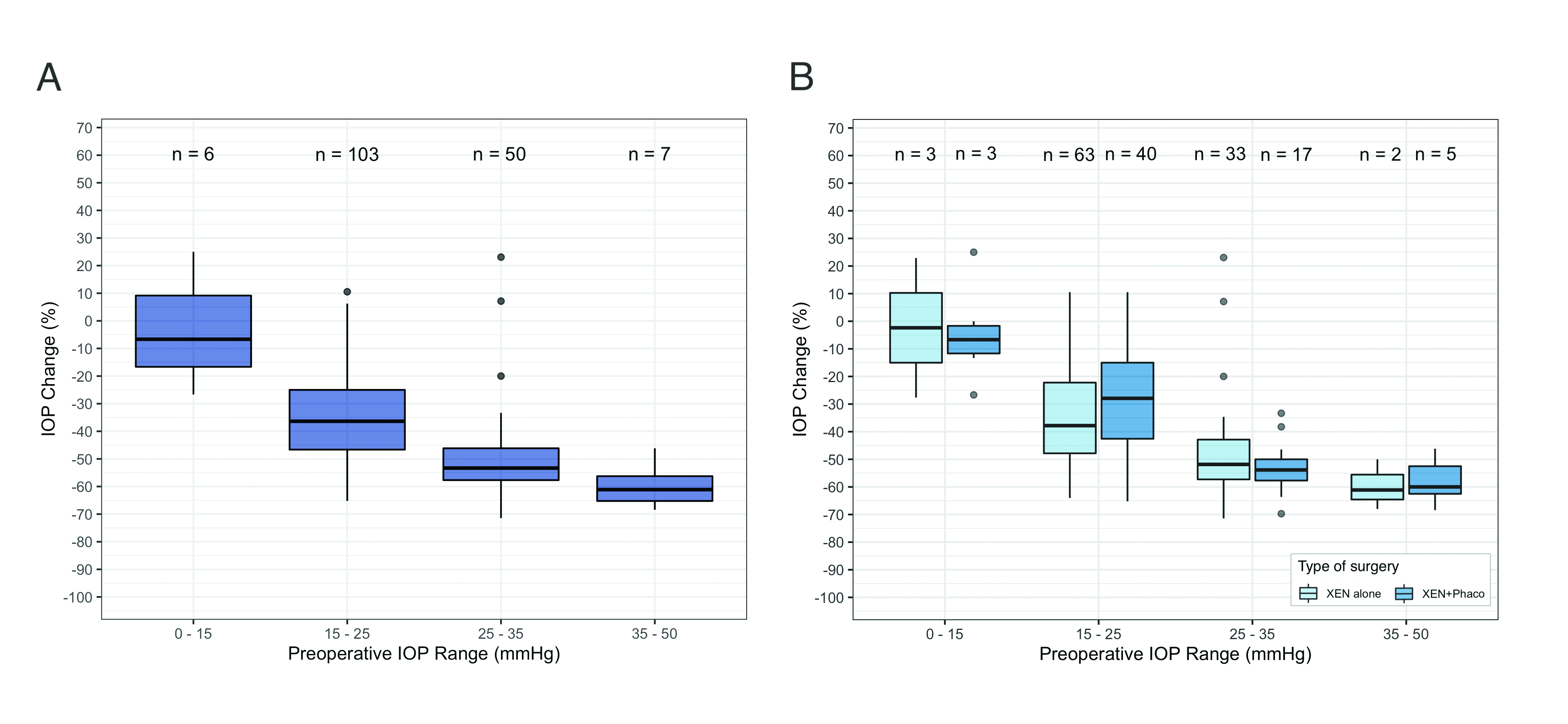

Supplement: Supplementary file 3 — Supplementary Figure 1 [file 41433_2023_2642_MOESM3_ESM.tif]

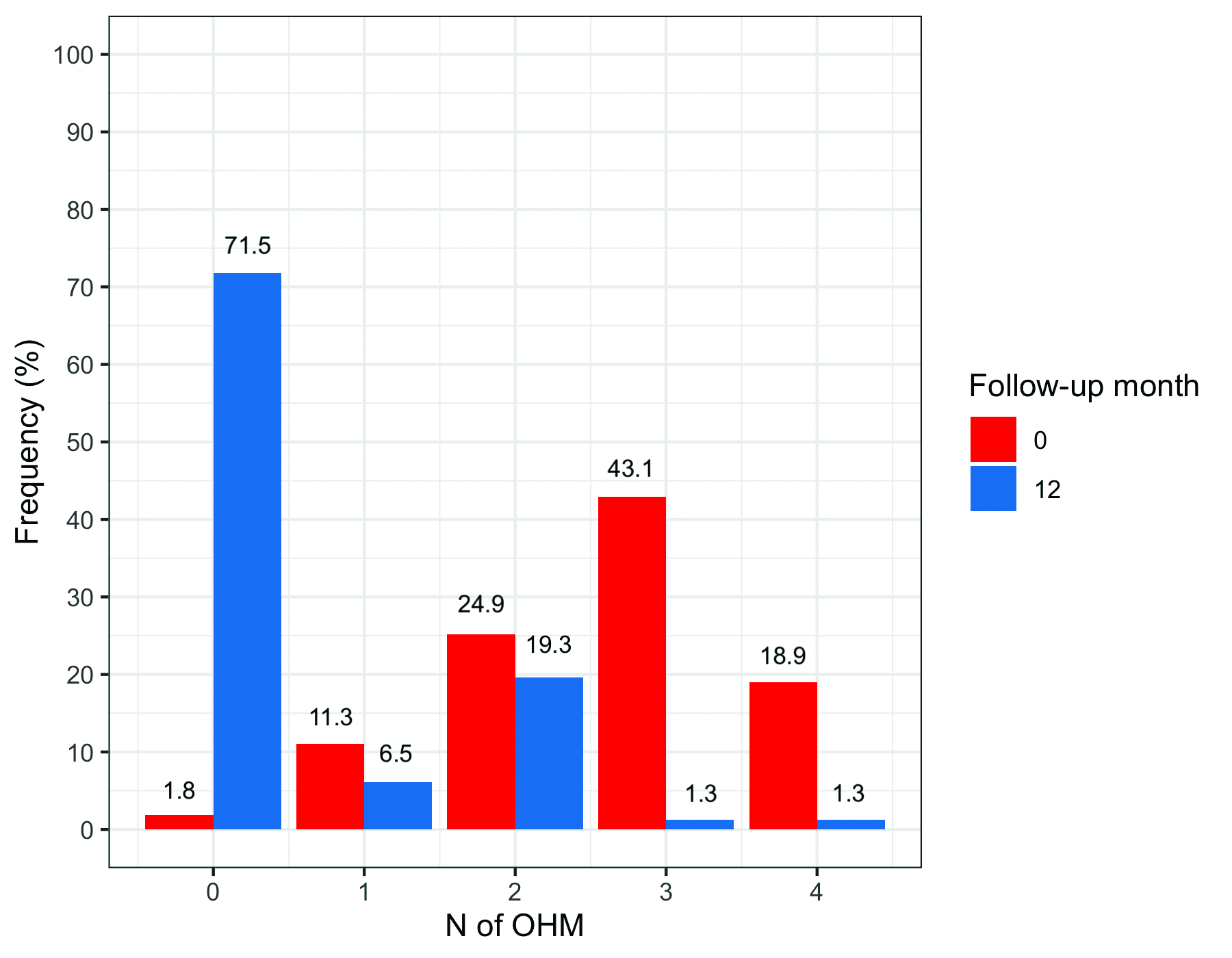

Supplement: Supplementary file 4 — Supplementary Figure 2 [file 41433_2023_2642_MOESM4_ESM.tif]

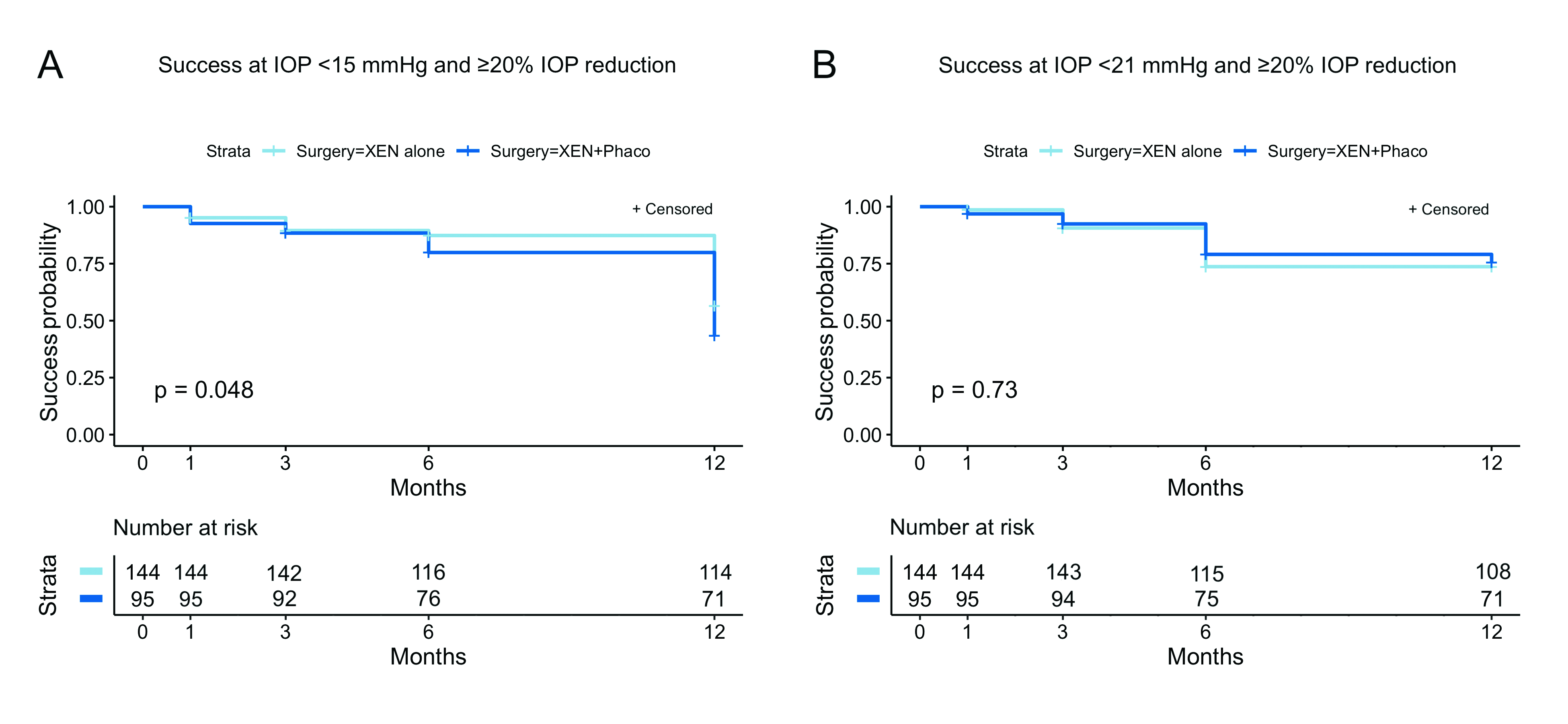

Supplement: Supplementary file 5 — Supplementary Figure 3 [file 41433_2023_2642_MOESM5_ESM.tif]
